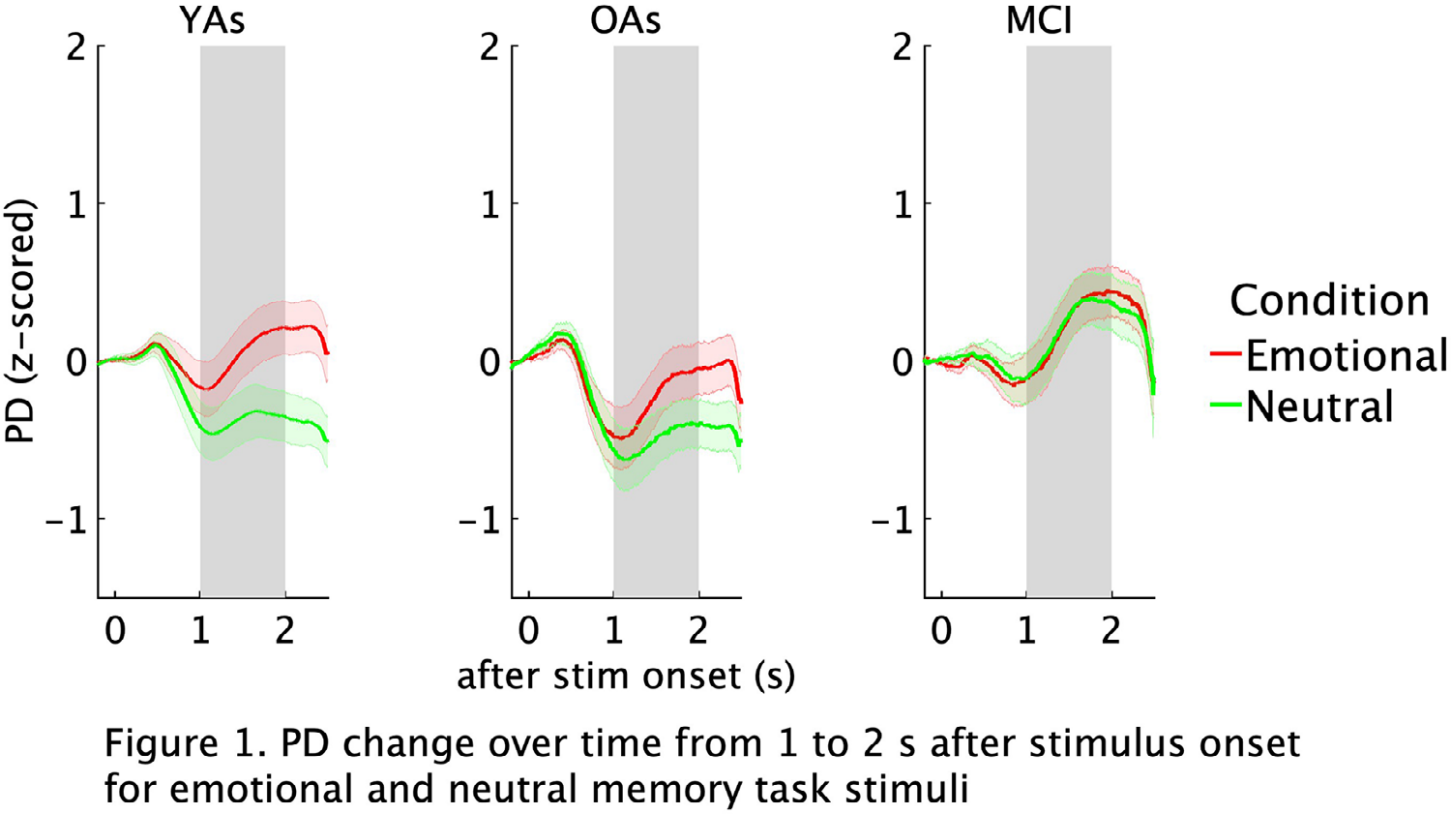# Changes in Task‐evoked Pupil Dilation during Emotional Memory as a Marker of Noradrenergic Function in Healthy Aging and MCI

**DOI:** 10.1002/alz70861_108410

**Published:** 2025-12-23

**Authors:** Alina Zhunussova, Clare Loane, Elif Kurt, Grazia Daniela Femminella, Sabrina Lenzoni, Millie Duckett, Martina F Callaghan, Nikolaus Weiskopf, Raymond J Dolan, Robert Howard, Emrah Düzel, Dorothea Hämmerer

**Affiliations:** ^1^ University of Innsbruck, Innsbruck Austria; ^2^ Institute of Cognitive Neuroscience, University College London, London UK; ^3^ Aziz Sancar Institute of Experimental Medicine, Istanbul University, Istanbul Turkey; ^4^ University of Naples Federico II, Napoli Italy; ^5^ University of Innsbruck, Innsbruck, Tyrol Austria; ^6^ Queen Square Institute of Neurology, University College London, London UK; ^7^ Wellcome Centre for Human Neuroimaging, Queen Square Institute of Neurology, University College London, London UK; ^8^ Max Planck Institute for Human Cognitive and Brain Sciences, Leipzig Germany; ^9^ Felix Bloch Institute for Solid State Physics, Faculty of Physics and Earth Sciences, Leipzig University, Leipzig Germany; ^10^ Max Planck University College London Centre for Computational Psychiatry and Ageing Research, University College London, London UK; ^11^ Division of Psychiatry, University College London, London UK; ^12^ Institute of Cognitive Neurology and Dementia Research (IKND), Otto‐von‐Guericke University, Magdeburg Germany; ^13^ German Center for Neurodegenerative Diseases (DZNE), Magdeburg Germany; ^14^ Center for Behavioral Brain Sciences, Magdeburg Germany; ^15^ German Center for Neurodegenerative Diseases, Magdeburg Germany; ^16^ Institute of Cognitive Neurology and Dementia Research, Otto‐von‐Guericke University Magdeburg, Magdeburg Germany

## Abstract

**Background:**

Improved memory for negative events is supported by the locus coeruleus‐noradrenergic (LC‐NA) system. As the LC is one of the first structures exhibiting tau pathologies in AD, in vivo markers for structural and functional changes in the LC are required. We used pupil dilation (PD) as a non‐exclusive and indirect but easy and non‐invasive way to acquire a measure of LC‐NA activity. We investigated whether PD can serve as a marker of alteration in noradrenergic function in mild cognitive impairment (MCI) during emotional memory.

**Method:**

The emotional memory task was completed by 83 participants (28 younger adults (YAs), 25 older adults (OAs), and 30 patients with MCI) during eye‐tracking recordings. In the incidental encoding task, participants were shown a neutral or negative indoor or outdoor scene and were instructed to categorize it as either indoor or outdoor. In the immediate recognition test and the delayed recognition test (6 hours later), participants were instructed to classify the pictures as new or old.

**Result:**

PD was larger for emotional as compared to neutral scenes in YAs and OAs but overall increased in MCI patients, regardless of scene type, suggesting a deficiency in emotional regulation. The increase in emotional reactivity in MCI does not translate into improved emotional memory; as expected, emotional scenes were better remembered than neutral scenes, with MCI patients showing worse memory than YAs or OAs.

**Conclusion:**

PD can be reliably measured in patients with MCI. Moreover, changes in task‐evoked PD during an emotional memory task can reveal differences between healthy aging and MCI in emotional reactivity.